# Characterization of a Novel Chimeric *Theileria parva* p67 Antigen Which Incorporates into Virus-like Particles and Is Highly Immunogenic in Mice

**DOI:** 10.3390/vaccines10020210

**Published:** 2022-01-28

**Authors:** Leah Whittle, Ros Chapman, Michiel van Diepen, Edward P. Rybicki, Anna-Lise Williamson

**Affiliations:** 1 Institute of Infectious Diseases and Molecular Medicine, University of Cape Town, Cape Town 7925, South Africa; WHTLEA002@myuct.ac.za (L.W.); michiel.vandiepen@watchmakergenomics.com (M.v.D.); ed.rybicki@uct.ac.za (E.P.R.); anna-lise.williamson@uct.ac.za (A.-L.W.); 2 Division of Medical Virology, Department of Pathology, Faculty of Health Sciences, University of Cape Town, Cape Town 7925, South Africa; 3 Biopharming Research Unit, Department of Molecular and Cell Biology, University of Cape Town, Cape Town 7925, South Africa

**Keywords:** East Coast Fever, *Theileria parva*, p67, Gag, virus-like particles, vaccine

## Abstract

The current method to protect cattle against East Coast Fever (ECF) involves the use of live *Theileria parva* sporozoites. Although this provides immunity, using live parasites has many disadvantages, such as contributing to the spread of ECF. Subunit vaccines based on the sporozoite surface protein p67 have been investigated as a replacement for the current method. In this study, two DNA vaccines expressing recombinant forms of p67 designed to display on retrovirus-like particles were constructed with the aim of improving immunogenicity. The native leader sequence was replaced with the human tissue plasminogen activator leader in both vaccines. The full-length p67 gene was included in the first DNA vaccine (p67); in the second, the transmembrane domain and cytoplasmic tail were replaced with those of an influenza A virus hemagglutinin 5 (p67HA). Immunofluorescent staining of fixed and live transfected mammalian cells showed that both p67 and p67HA were successfully expressed, and p67HA localised on the cell surface. Furthermore, p67HA was displayed on the surface of both bovine leukaemia virus (BLV) Gag and HIV-1 Gag virus-like particles (VLPs) made in the same cells. Mice vaccinated with DNA vaccines expressing p67 and p67HA alone, or p67HA with BLV or HIV-1 Gag, developed high titres of p67 and BLV Gag-binding antibodies. Here we show that it is possible to integrate a form of p67 containing all known antigenic domains into VLPs. This p67HA–VLP combination has the potential to be incorporated into a vaccine against ECF, as a DNA vaccine or as other vaccine platforms.

## 1. Introduction

Of all the tick-borne diseases affecting cattle in Africa, East Coast Fever (ECF) is considered one of the most concerning due to its severity and economic burden in the east and sub-Saharan regions. The disease affects 12 countries and can have high incidence and mortality rates; regions in Uganda have case fatality rates of up to 89.5% [1,2]. It was also recently detected in Cameroon for the first time [3]. In Tanzania it is estimated to kill up to one million cattle every year and to cause a total loss of approximately US$ 247 million annually [4]. Compared to other tick-borne diseases, ECF was ranked first in importance by pastoralists in many countries, including Kenya, Grande Comore, Tanzania and regions in South Sudan [5,6,7,8]. The disease is caused by *Theileria parva,* a eukaryotic apicomplexan parasite that is spread to both cattle and buffalo by the brown ear tick *Rhipicephalus appendiculatus* [9]. The venerable infection and treatment method (ITM) is the current vaccination regime against ECF: cattle are infected with live *T. parva* sporozoites and treated immediately afterwards with long-acting oxytetracycline [10]. This provides effective protection but has many disadvantages: these include the use of cattle, rabbits and ticks for the generation and extensive quality control of vaccine sporozoites; transport of live parasites in liquid nitrogen, which requires a cold chain, and the fact that the vaccine is lethal if the appropriate antibiotic dosage is not administered [2,11]. Furthermore, ITM-vaccinated cattle become *T. parva* carriers, which can spread the parasite to unvaccinated animals and introduce ECF into previously naïve regions [12,13].

A novel vaccine that can replace the time-consuming and laborious ITM procedure is therefore desirable. In attempts to achieve this, numerous groups have investigated the *T. parva* major sporozoite surface protein p67 for use as a subunit vaccine [14]. The antigen is conserved across cattle-derived *T. parva* strains and can induce the production of sporozoite-neutralising antibodies [15]. It is thought to aid in the attachment and invasion of the sporozoite into host lymphocytes, although the mechanism is unknown [16]. This protein is also expressed at a high level in the sporoblast and at a very low level in the schizont [17]. Wildtype p67 consists of a signal sequence (SS), a predicted transmembrane domain (TM) and one predicted cytoplasmic residue [18]. Others have separated p67 into three domains; namely, p67N (N-terminal region), p67M (middle region) and p67C (C-terminal region) based on B-cell epitope distribution, with known sporozoite neutralising epitopes present in p67N and p67C [15,19]. Sera from rats inoculated with p67M have been shown to neutralise sporozoites; however, this domain of p67 excluding p67N and p67C has not been investigated in depth or tested in cattle [20].

Expression of complete p67—containing all three p67N, p67M and p67C domains—in bacteria, insect cells and mammalian cell lines results in an unstable protein [20,21,22]. Different forms of full length p67 or p67 lacking the SS, TM or both consistently appear as a range of protein sizes on western blots. Why these occur is unknown, although these forms are highly immunogenic in animals and confer partial protection against *T. parva* in cattle. Animals inoculated with p67ΔTM produced in mammalian cells can produce p67-binding antibodies, and those vaccinated with three doses of bacterially-produced p67 lacking the SS and TM in adjuvant were partially protected from *T. parva* challenge (57% protection) [22,23].

Truncated versions of p67, and specifically p67C, appear to be more stable than the full-length version and still provide partial protection against ECF. In contrast to the larger forms of the protein, p67C appears as one predominant size on western blots [24]. Three doses of soluble p67C with adjuvant gave partial protection from *T. parva* challenge in cattle (46% protection) [25]. Protection was correlated with p67 antibody titres and CD4+ proliferation.

The immunogenicity of p67C can be further improved by displaying the antigen on the surface of a virus or nanoparticle. Mice inoculated with baculovirus-displayed p67C showed a >25-fold increase in p67-binding antibody titres compared to soluble p67C [26]. Likewise, sera from cattle inoculated with p67C conjugated to silica vesicles or displayed on hepatitis B virus-like particles (VLPs) had increased sporozoite neutralisation capabilities in comparison to soluble p67C [27].

VLPs are self-assembling nanoparticles of viral origin that enable the highly ordered display of antigens on the VLP surface and have been used extensively in vaccines to augment immunogenicity [28]. It may be that presenting antigens that are normally found anchored onto the surface of pathogens on VLP surfaces may be beneficial, as the antigen might be better stabilised in a more native conformation compared to soluble protein. Both humoral and cellular responses can be significantly improved compared to using free antigens [29,30].

Displaying a full length form of p67 on the surface of a VLP may be advantageous, as it would include all the known immunogenic regions of the protein. A GFP–p67 chimera without the native SS and TM has been shown to induce significantly higher antibody titres in mice compared to the more truncated GFP–p67N and GFP–p67C forms [26]. This unanchored soluble GFP–p67 chimaera also appeared to provide better protection than baculovirus-displayed p67C [31]. Thus, it may be possible to even further improve p67-based vaccines by displaying less-truncated forms of p67 on a VLP.

Integration of antigens into enveloped VLPs, such as those based on the retrovirus Gag protein, requires the antigen to be present at the plasma membrane with a transmembrane domain [32]. Although p67 contains a predicted C-terminal TM and can be found on the surface of *T. parva* sporozoites, targeting the protein to the cell surface of insect and various mammalian cell lines has been challenging [18,21,33]. Association of p67 with Gag-based VLPs would require modification, such as the addition of a different membrane anchor to enable presentation at the cell surface. The TM of glycoproteins from different viruses can interact with human immunodeficiency virus (HIV) Gag to form VLPs. Fusion of the HIV envelope glycoprotein (Env) to the TM and cytoplasmic tails (CT) of other viral surface proteins, such as mouse mammary tumour virus (MMTV), influenza A hemagglutinin 2 domain (HA_2_) and baculovirus gp64, still allows the incorporation of Env into Gag-based VLPs [34,35,36].

Here, we investigated the engineering of a novel p67 chimera for both plasma membrane localisation and Gag-VLP incorporation of all known antigenic domains, with the aim of improving the expression and immunogenicity of the ECF antigen. The predicted p67 TM and CT sequences were replaced with those of influenza HA_2_, and expression was compared to p67 with the native TM and CT by expression in mammalian cells. Incorporation into bovine leukaemia virus (BLV) Gag and HIV Gag VLPs was characterised, and the immunogenicity of the DNA constructs was assessed in mice. BLV is another important bovine pathogen, affecting cattle globally [37]. We show that BLV Gag VLPs can be used for heterologous antigen display. Use of BLV Gag VLPs could be advantageous as it may also provide immune responses and protection against BLV.

## 2. Materials and Methods

### 2.1. Plasmids, Cells and Primary Antibodies

**Figure 1:**
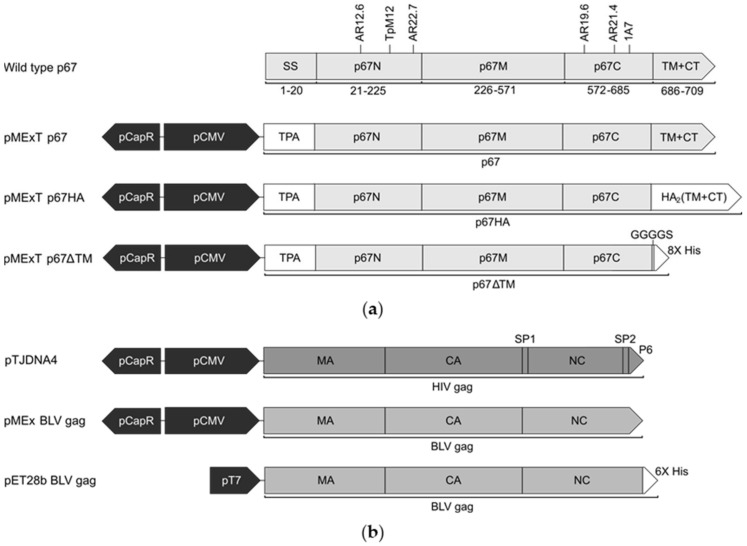
Schematic diagrams of the expression cassettes used in this study. (**a**) Wild type Muguga *T. parva* p67 (GenBank: M67476.1) with annotated amino acid residue positions, signal sequence (SS), N-terminal (p67N), middle (p67M) and C-terminal (p67C) domains, transmembrane domain (TM), cytoplasmic tail (CT) and the position of neutralising epitopes recognised by AR12.6, TpM12, AR22.7, AR19.6, AR21.4 and 1A7 murine antibodies. The modified p67 constructs are shown with annotated regions: porcine circovirus 1 enhancer (pCapR), cytomegalovirus I/E promoter (pCMV), human tissue plasminogen activator leader sequence (TPA), influenza hemagglutinin 2 (HA_2_) TM and CT, Gly Gly Gly Gly Ser linker (GGGGS) and 8x poly-histidine tag (8x His). (**b**) Schematic diagrams of expression cassettes encoding Gag with annotated domains: matrix (MA), capsid (CA), nucleocapsid (NC) and spacer (SP). All pMEx vectors and pTJDNA4 [41] are based on the mammalian expression vector pTHpCapR [39]. The bacterial expression vector pET28b BLV gag with the bacteriophage T7 RNA polymerase promoter (pT7) encodes for BLV gag fused to a 6x poly-histidine tag (6x His).

The p67 sequence used in this study was based on the *T. parva* cattle Muguga strain (GenBank: M67476.1) (Figure 1a). The p67N, p67M and p67C domains and murine B-cell epitopes are annotated as previously described [15,24], and the signal peptide and TM predicted by Phobius, similarly to Tebaldi, Williams [22,38].

The mammalian expression vectors (Figure 1) are based on the plasmid backbone pTHpCapR, which contains a porcine circovirus 1 (PCV-1) enhancer in the reverse orientation upstream of the cytomegalovirus intermediate/early promoter (pCMV), enabling very high expression of inserted genes [39]. The backbone is referred to as pMEx or pMExT in this study: plasmid for mammalian expression with the human tissue plasminogen activator (TPA) signal sequence [40]. The p67 gene was codon-optimised for cattle expression and synthesized by GenScript (China). Plasmids encoding p67 have the native signal sequence replaced with that of TPA (GenBank: CAX11668.1) to aid entry to the secretory pathway. pMExT p67 encodes p67 from amino acid residue 21 up to the single cytoplasmic residue. pMExT p67HA has the p67N, p67M and p67C domains fused to the TM and CT of influenza A H5N1 hemagglutinin 2 (HA_2_) (GenBank: NC_007362.1). pMExT p67ΔTM encodes p67N, p67M and p67C fused to a GGGGS linker and eight histidine residues to enable purification of the expressed soluble protein. pMEx BLV gag encodes BLV Gag (GenBank: AP018021.1) codon-optimised for cattle expression, synthesised by GenScript (China). The BLV gag gene was subcloned from pMEx BLV gag into the pET28b bacterial expression vector to construct pET28b BLV gag. pTJDNA4 encodes HIV-1 subtype C mosaic Gag and also has pMEx as the plasmid backbone [41]. All DNA plasmids used for mouse vaccinations were produced by GenScript (China).

HEK293T cells (CRL-3216, ATCC, Manassas, Virginia, USA) were cultured in Dulbecco’s Modified Eagle Medium (high glucose, GlutaMAX™) (Thermo Fisher Scientific, USA) with 1× penicillin/streptomycin (Lonza, Belgium) and 10% foetal bovine serum (Thermo Fisher Scientific, Waltham, Massachusetts, USA).

Polyclonal rabbit anti-p67 raised against the peptide LKKTLQPGKTSTGETC, which contains the neutralising epitope LQPGKTS recognised by AR22.7 (Figure 1a) [15], was produced by GenScript (China). Mouse anti-BLV-p24 was used to detect BLV Gag (BLV3, VMRD, Pullman, Washington, USA) and mouse anti-histidine (MCA1396, Bio-Rad, Hercules, California, USA) to detect tagged-p67ΔTM.

### 2.2. Confirmation of p67, p67HA and BLV Gag Expression

Immunofluorescent staining of live and fixed HEK293T cells in 12-well plates transfected with 1 µg/well of DNA using 1 µL XtremeGENE HP (Roche, Basel, Switzerland) was performed as previously described [40]. For live staining, cells were incubated with primary and secondary antibodies for 1 h each. To detect p67 and p67HA, anti-p67 was used at 1:2000 and donkey anti-rabbit-IgG Cy3 (red) at 1:1000 (Life Technologies, Carlsbad, California, USA); to detect BLV Gag, anti-BLV p24 was used at 1:2000 and donkey anti-mouse-IgG Alexa Fluor 488 (green) at 1:1000 (Life Technologies, USA). Cells were viewed and imaged with an AxioVertA.1 inverted fluorescent microscope (Zeiss, Germany) and processed with Zen Blue 3.1 software (Zeiss, Germany).

To confirm expression by western blotting, HEK293T cells in 12-well plates were transfected with 1 µg/well of the DNA using 1 µL XtremeGENE HP (Roche, Switzerland). After 3 days, the media were collected, cells were lysed with 200 µL Glo Lysis buffer (Promega, Madison, Wisconsin, USA) and the samples were clarified by centrifugation (10 min at 9660× *g*). Samples were mixed with Laemmli buffer, boiled for 5 min at 95 °C, 15 µL per lane was loaded and samples were separated using denaturing SDS PAGE and transferred to a polyvinylidene difluoride (PVDF) membrane (Bio-Rad, USA). The membranes were probed with rabbit anti-p67 (1:5000) and anti-BLV Gag (1:5000), and secondary antibodies goat anti-rabbit-IgG (A3687, Sigma, Burlington, Massachusetts, USA) or goat anti-mouse-IgG (A3562, Sigma, USA) were conjugated to alkaline phosphatase, both at 1:10,000 and detected with 5-bromo-4-chloro-3-indolylphosphate (BCIP)/nitroblue tetrazolium (NBT) phosphatase substrate (KPL, Seracare Life Sciences, Gaithersburg, Maryland, USA).

### 2.3. VLP Isolation, Immunogold-Labelling and Electron Microscopy

VLPs were isolated using a similar method to that described elsewhere [35]. T75 flasks of HEK293T cells were transfected with 30 µg polyethylenimine (PEI) (Sigma, USA), 20 µg of pMExT p67 or pMExT p67HA and co-transfected with 10 µg of either pMEx BLV gag or pTJDNA4. Cells were also transfected with separate plasmids as controls. At 3 days’ post-transfection, media were clarified by centrifugation at 1260× *g* for 10 min. Supernatants were underlaid with 5 mL 12% (*v*/*v*) OptiPrep (Sigma, USA) Tris-buffered saline (TBS) cushions in SS34 tubes and centrifuged at 47,807.6× *g* for 1 h at 4 °C. Pellets were resuspended in 100 µL ice-cold TBS and placed on glow-discharged carbon-coated copper grids for 30 s. Grids were blocked with 1% (*w*/*v*) bovine serum albumin (BSA) diluted in TBS for 1 min, washed thrice with TBS and incubated in rabbit anti-p67 diluted 1:500 in 0.1% (*w*/*v*) BSA/TBS for 2 h at 4 °C. These were washed thrice in 1% (*w*/*v*) BSA/TBS and incubated with goat anti-rabbit-IgG conjugated to 10 nm colloidal gold (G7402, Sigma, USA) diluted 1:50 in 0.1% (*w*/*v*) BSA/TBS for 30 min. Grids were washed thrice with TBS, once with H_2_O, twice with 2% uranyl acetate and then incubated with 2% uranyl acetate for 1 min. The VLPs were viewed by conventional transmission electron microscopy (TEM) with a Tecnai T20 microscope (FEI, Hillsboro, Oregon, USA).

### 2.4. Characterization of p67ΔTM Protein

To confirm expression of p67ΔTM, HEK293T cells in 12-well plates were transfected with pMExT p67ΔTM and with pMExT p67 as a control (1 µg DNA/well and either 1 µg XtremeGENE HP (Roche, Switzerland) or 3 µL PEI). Media and cell lysate were collected 3 days later with Glo Lysis buffer (Promega, USA), as described earlier. Samples were processed and used for denaturing SDS PAGE as described. Blots were probed with mouse anti-His (1:2000) or rabbit anti-p67 (1:5000), and goat anti-mouse-IgG (ab97020, Abcam, Cambridge, UK) or goat anti-rabbit-IgG (A3687, Sigma, USA) secondary antibodies conjugated to alkaline phosphatase, both at 1:10,000.

### 2.5. Purification of p67ΔTM and BLV Gag Protein

Soluble p67ΔTM and BLV Gag were purified via their His-tags to have protein for the detection of binding antibodies in mouse serum.

HEK293T cells in eight T175 flasks were transfected with 40 µg of pMExT p67ΔTM using 120 µL PEI per flask. After 3 days, media were clarified by centrifugation at 1260× *g* for 10 min, supernatants were pooled and HEPES (Thermo Fisher Scientific, USA) was added at a final concentration of 10 mM for pH stabilisation.

To produce BLV Gag, BL21 CodonPlus DE3 RIL bacteria (Agilent Technologies, Santa Clara, California, USA) transformed with pET28b BLV gag were cultured in 500 mL of Luria broth with 50 µg/mL kanamycin (Thermo Fisher Scientific, USA) and 35 µg/mL chloramphenicol (Sigma, USA). The culture was induced with 0.6 mM IPTG at OD_600_ 0.582 overnight at room temperature. Cells were pelleted at 10,000× *g* for 10 min and resuspended in 25 mL 1× cOmplete EDTA-free protease inhibitor (Roche, Switzerland) diluted in phosphate-buffered saline (PBS). Lysis was performed by sonication on ice and insoluble material was pelleted by centrifugation at 10,000× *g* for 5 min.

Purification of p67ΔTM from clarified HEK293T cell media and BLV Gag from clarified bacterial lysate were both performed according to a previously described protocol [42]. The supernatant or lysate was added to activated cobalt agarose resin (HisPur™ Cobalt Superflow Agarose, Thermo Fisher Scientific, USA) in 10 mL syringe columns with glass wool: HEK293T supernatant was added to 10 mL of resin settled in the column, whereas bacterial lysate was incubated with 10 mL resin for 1 h on ice with shaking prior to loading of the mixture into the syringe. The columns were washed with binding buffer (wash 1) and wash buffer (wash 2) and proteins were eluted with elution buffer. For p67ΔTM, purification took place at 4 °C with a peristaltic pump (Gilson Minipuls 3), whereas BLV Gag was purified at room temperature by gravity flow. Protein concentration and buffer exchange from elution buffer to TBS (p67ΔTM) or PBS (BLV Gag) were performed using concentration columns (Vivaspin 20 30 kDa MWCO, GE Healthcare, North Richland Hills, Texas, USA) to have a final volume of 1 mL purified protein. Protein concentrations were determined by a DC assay (Bio-Rad, USA).

Purification of p67ΔTM and BLV Gag was confirmed by SDS PAGE and Coomassie Blue staining (stained for 1 h in staining solution (0.125% (*w*/*v*) Coomassie Blue G-250 (Bio-Rad, USA), 50% (*v*/*v*) methanol and 10% (*v*/*v*) acetic acid), de-stained overnight with de-staining solution (50% (*v*/*v*) methanol and 10% (*v*/*v*) acetic acid) and soaked in H_2_O) (Figure S1).

### 2.6. Mouse Immunizations

Female BALB/c mice at the University of Cape Town (UCT) Research Animal Facility were used to determine the immunogenicity of the DNA vaccines. All protocols were approved by the UCT Animal Ethics Committee (AEC 019-018). Groups of mice, 5 per group, were inoculated intramuscularly once every two weeks for a total of four times with 100 µg pMExT p67 (p67), 100 µg pMExT p67HA (p67HA), 100 µg pMExT p67HA and 100 µg pMEx BLV gag (p67HA + BLV gag), 100 µg pMExT p67HA and 100 µg pTJDNA4 (p67HA + HIV gag) or PBS (Thermo Fisher Scientific, USA) as a negative control. All vaccines were diluted in PBS. End-bleeds were performed by cardiac puncture 10 days after the last vaccination.

### 2.7. ELISAs for p67- and BLV Gag-Binding Antibodies

ELISA plates were incubated overnight at 4 °C with 100 ng per well of purified protein in PBS: Pierce nickel-coated 96-well ELISA plates (Thermo Fisher Scientific, USA) were coated with p67ΔTM and Nunc MaxiSorp 96-well ELISA plates (Thermo Fisher Scientific, USA) were coated with BLV Gag. Wells were washed thrice with PBS and blocked with blocking buffer (5% (*w*/*v*) skim milk in PBS) for 1 h at room temperature. Plates were washed thrice with PBS and incubated with sera diluted 3-fold in 2.5% (*w*/*v*) skim milk in PBS. These were washed thrice with PBST (PBS with 0.1% (*v*/*v*) Tween 20), then incubated with goat anti-mouse-IgG conjugated to horseradish peroxidase (HRP) (ab97023, Abcam, UK), 1:10,000, for 1 h at room temperature. Plates were washed thrice with PBST, incubated with tetramethylbenzidene (Abcam, UK) for 7 min (p67ΔTM) or 10 min (BLV Gag) and the reactions stopped with 1 N H_2_SO_4_. Readings were taken at 450 nm subtracted by 540 nm to remove background (VersaMax ELISA Microplate Reader, Molecular Devices, Silicon Valley, California, USA). Endpoint titres for each mouse were determined by taking the reciprocal of the highest dilution that had a reading 2-fold greater than that of the average background PBS control group sera at the lowest dilution. A one-way ANOVA with a post-hoc Bonferroni test was conducted using Prism version 5.0 (GraphPad, San Diego, California, USA).

## 3. Results

### 3.1. Confirmation of p67, p67HA and BLV Gag Expression

**Figure 2:**
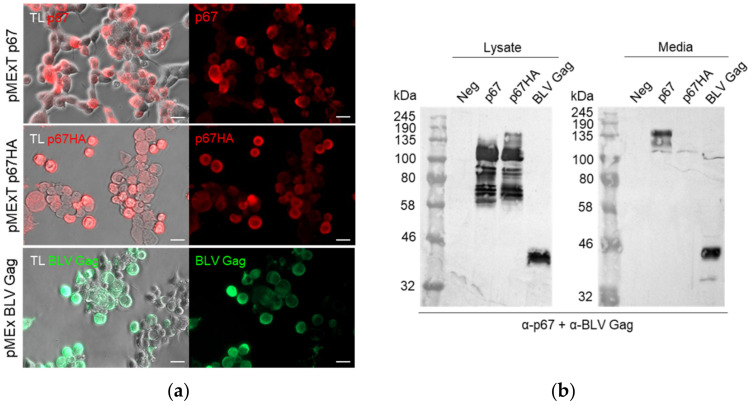
Confirmation of p67, p67HA and BLV Gag expression. (**a**) HEK293T cells were transfected with pMExT p67, pMExT p67HA or pMEx BLV gag, fixed after 3 days and probed with α-p67 and α-rabbit-IgG Cy3 (red) or α-BLV Gag and α-mouse-IgG Alexa Fluor 488 (green). Fluorescence with transmitted light (TL) is shown as well. Scale bars: 20 µm. (**b**) SDS PAGE and western blotting. Clarified lysates (left) and media (right) were collected from HEK293T cells transiently transfected with no DNA (Neg), pMExT p67 (p67), pMExT p67HA (p67HA) or pMEx BLV gag (BLV Gag). Blots were probed with both α-p67 and α-BLV Gag.

DNA plasmids were constructed to express p67 and p67HA: both pMExT p67 and pMExT p67HA had the human TPA leader in place of the native p67 signal sequence, and pMExT p67HA had the native transmembrane domain and cytoplasmic tail replaced with that of influenza HA_2_ (Figure 1a). The DNA plasmid pMEx BLV gag encoded unmodified BLV Gag for VLP formation (Figure 1b). Expression of p67, p67HA and BLV Gag was confirmed by immunofluorescent staining of fixed HEK293T cells and western blotting (Figure 2). Proteins ranging in size from approximately 58 kDa to 140 kDa were seen for p67 and p67HA cell lysate samples on western blots (Figure 2b), which is characteristic of recombinant p67 [22].

p67 was observed to migrate as 110 kDa to 140 kDa proteins in the media sample. Except for a faint 110 kDa product, these larger proteins were absent for p67HA in the media, although they were detected in the lysate. BLV Gag (44 kDa) was detected as expected in both the cell lysate and media.

### 3.2. Presence of p67HA on the Cell Surface and Isolated VLPs

**Figure 3:**
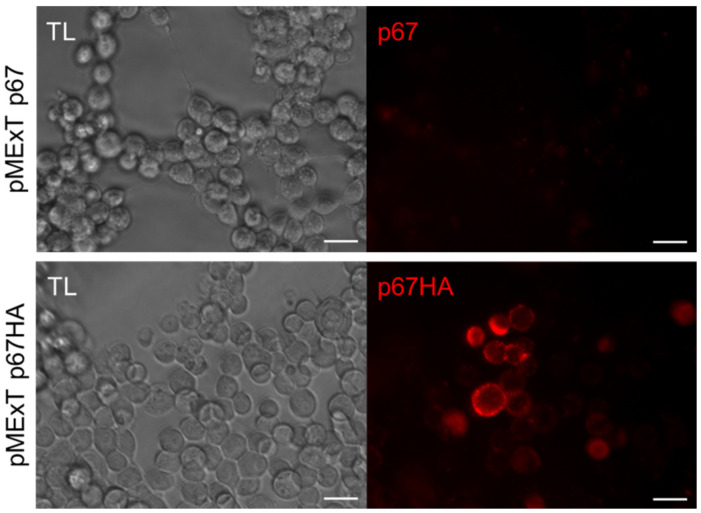
Detection of p67HA on the cell surface. HEK293T cells were transfected with pMExT p67 or pMExT p67HA and live-stained after three days with α-p67 and α-rabbit-Cy3 (red). Scale bars: 20 µm.

VLP-display of antigens can potentially enhance their immunogenicity [30], hence BLV Gag and HIV Gag VLPs were used for this purpose. Plasma membrane localisation is required for incorporation of antigens into Gag VLPs, therefore live-cell staining was performed to determine if expressed p67 and p67HA were present at the cell surface (Figure 3). This method would not detect intracellular protein but only protein on the surface of live cells. A clear difference in localisation could be seen between the two proteins—p67 showed very little fluorescence with no specific pattern of expression, whereas cells expressing p67HA fluoresced strongly, indicating that p67HA is present on the cell surface.

**Figure 4:**
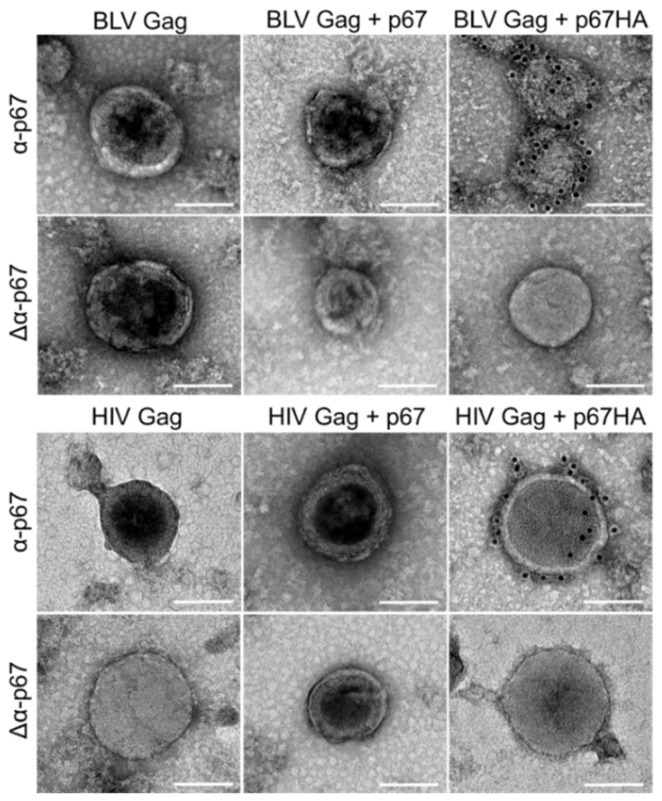
Immunogold-labelling of p67HA on the surface of BLV Gag VLPs and HIV Gag VLPs isolated from transfected HEK293T cells. Grids were probed with α-p67 or with secondary antibody only (Δα-p67). VLPs were viewed by conventional TEM. Scale bar: 100 nm.

Immunogold-labelling experiments were performed on isolated VLPs to determine if p67 and p67HA can be displayed on the VLP surface (Figure 4). Isolated HIV Gag VLPs appeared as previously observed from pTJDNA4 [35]. BLV Gag VLPs were seen, demonstrating that VLPs can assemble when expressed from pMEx BLV gag. As expected, HIV Gag and BLV Gag VLPs co-expressed with p67 showed no labelling of the antigen. However, both HIV Gag and BLV Gag VLPs expressed with p67HA showed gold labelling at high densities. Exclusion of the primary α-p67 antibody (Δα-p67) resulted in no surface labelling of p67HA. No VLPs were seen in samples prepared from HEK293T cells transfected with no DNA.

### 3.3. Characterisation of p67ΔTM Expression

**Figure 5:**
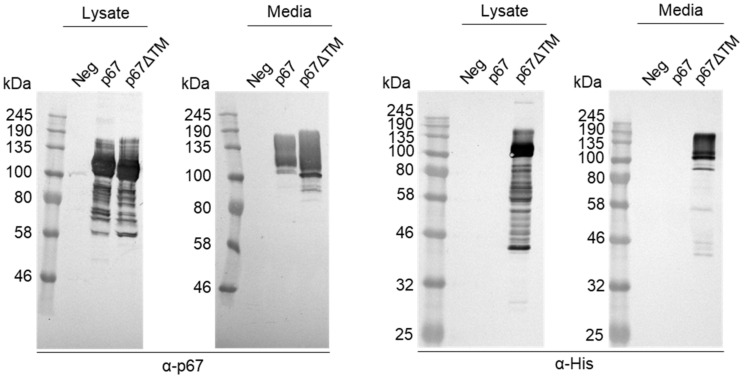
Confirmation of p67ΔTM expression by SDS PAGE and western blotting. Clarified lysates and media were collected from HEK293T cells transiently transfected with no DNA (Neg), pMExT p67 (p67) or pMExT p67ΔTM (p67ΔTM). Blots were probed with α-p67 (left) or α-His (right).

A soluble His-tagged form of p67 named p67ΔTM was created to provide purified protein for the detection of p67-binding antibodies in animal sera. Expression of p67ΔTM in HEK293T cells was confirmed by western blotting (Figure 5). Like p67 and p67HA, p67ΔTM was also detected as a range of differently sized proteins when probed with α-p67. Interestingly, p67ΔTM in the media gave a larger smear as well as smaller proteins. Presence of the His tag was confirmed by probing with α-His and showed smaller products ranging from under 46 kDa to 58 kDa which were not detected by α-p67. The His-tag is adjacent to p67C whereas α-p67 recognises the AR22.7 epitope near the end of p67N, therefore it is possible that α-His detected truncated or degraded products that did not contain p67N. p67 was not detected with α-His due to the absence of a His-tag.

### 3.4. Immunogenicity of p67, p67HA and p67HA-VLPs

**Figure 6:**
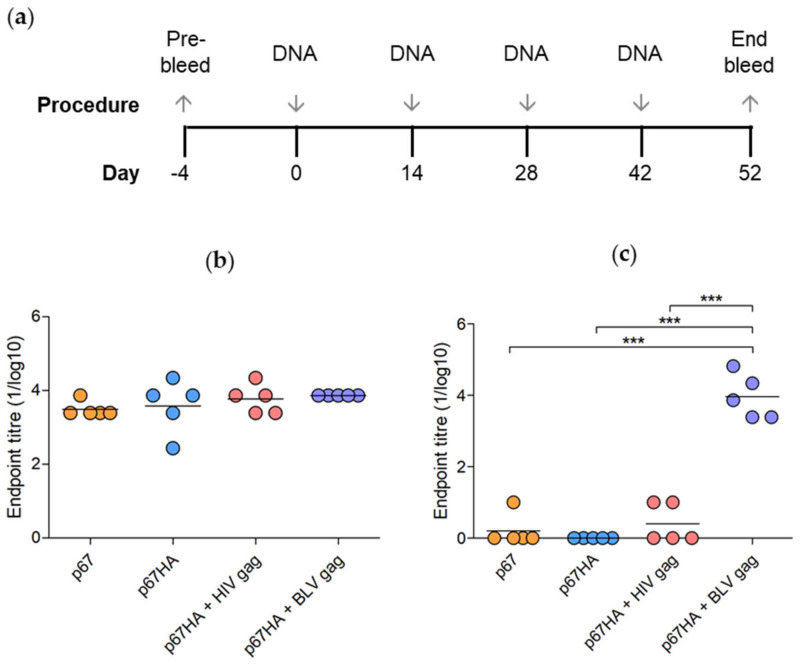
Vaccination regime and detection of binding antibodies in mice inoculated with DNA vaccines. (**a**) Mice were inoculated four times with 100 µg pMExT p67 (p67), 100 µg pMExT p67HA (p67HA), 100 µg pMExT p67HA and 100 µg pTJDNA4 (p67HA + HIV gag), 100 µg pMExT p67HA and 100 µg pMEx BLV gag (p67HA + BLV gag) or PBS as a negative control. Serum was collected at day 52 at the end bleed. p67-binding (**b**) and BLV Gag-binding (**c**) antibodies in mouse sera were detected by ELISAs. Each symbol represents an endpoint titre (1/log10) for an individual mouse and the lines represent endpoint means of each group. Responses below the PBS background were set to zero. *** *p* < 0.0001, one-way ANOVA with post hoc Bonferroni test.

To assess the immunogenicity of the DNA vaccines, mice were injected with pMExT p67, pMExT p67HA, pMExT p67HA + pMEx BLV gag, pMExT p67HA + pTJDNA4 or PBS (negative control) and ELISAs were performed using the mouse serum to detect the presence of p67- and BLV Gag-binding antibodies (Figure 6a). Antibodies that bind to HIV Gag were not investigated as HIV is not relevant for cattle vaccines.

All the mice in the experimental groups developed high titres of p67-binding antibodies (Figure 6b). There were no significant differences between the groups, however mice vaccinated with DNA vaccines expressing both p67HA and Gag developed higher titres of antibodies than those inoculated with plasmids expressing p67 or p67HA alone. Addition of BLV Gag appeared to result in more consistent titres compared to the other groups.

BLV Gag-binding antibodies were also detected in sera from mice that received pMExT p67HA + pMEx BLV gag (Figure 6c). Low, possibly non-specific titres of BLV Gag-binding antibodies were observed in the sera of mice vaccinated with p67 and p67HA + HIV Gag, however, these were significantly lower than those observed for mice inoculated with p67HA + BLV Gag. Results were negative for all mice that received PBS. As this was a pilot study, it should be noted that only five mice per group were assessed, thus further studies with larger numbers of animals need to be carried out to verify these results.

## 4. Discussion

The current ITM vaccination regime against ECF is problematic and can cause the spread of the disease to previously unaffected regions [12]. A novel vaccine that does not involve the use of live parasites is therefore desperately needed. While soluble p67 appears to be a suitable candidate ECF vaccine antigen, it does not fully protect from *T. parva* challenge. Recent partially successful challenge studies used truncated forms of p67 in their vaccines [25,27]. Therefore, our research aimed to improve the immunogenicity of p67 by displaying it on the surface of VLPs. p67 was modified by replacing the TM and CT with the corresponding regions from the influenza H5N1 hemagglutinin A2 (HA_2_). This enabled the successful display of p67HA at high density on the surface of BLV or HIV Gag VLPs. We showed that p67HA, but not p67, anchors to the cell surface of HEK293T cells and is incorporated into both BLV Gag- and HIV Gag-based VLPs. Furthermore, our DNA vaccines expressing p67HA alone or together with either BLV Gag or HIV Gag, were highly immunogenic in mice.

Previous work that characterised p67 showed the antigen was expressed as a range of protein sizes up to 140 kDa, in contrast to p67 extracted from *T. parva* sporozoites, which appeared as a predominant 67 kDa protein [21,22,43]. Tebaldi, Williams [22] demonstrated that p67ΔTM was secreted by mammalian cells as a 140 kDa protein that may be a dimer which only dissociated under severe denaturing conditions. Larger forms of p67ΔTM in cell media were also observed when expressed in insect cells [44]. Very similar 110–140 kDa forms were seen for our p67 and p67ΔTM in the media, whereas smaller proteins ranging from approximately 58–110 kDa present in the cell lysate were absent in the media (Figure 2b and Figure 6). These data may support the hypothesis that the secreted form of p67 aggregates or dimerizes, whereas smaller or monomeric forms are mostly retained inside the cell [22,44].

Even though our p67 with the native TM is transported out of the cell, it does not anchor to the plasma membrane (Figure 3). This agrees with earlier experiments where attempts at cell surface localisation of p67 met with difficulties. Full-length p67 with the native SS and TM does not express on the surface of insect cells [21]. These results interestingly conflict with the findings shown by Tebaldi et al. [22], where it was demonstrated by flow cytometry that their full p67 was present on the surface of HEK293T cells. This may be due to the different methods used to show p67 surface localisation. Others have shown that fusion of truncated forms of p67 to regions of baculovirus gp34 results in cell surface expression and display of the chimeric antigens on baculovirus virions [24]. Here, we show that replacing the native TM with that of HA_2_ results in anchorage to the cell membrane, as seen with p67HA (Figure 3). The 140 kDa secreted protein observed for p67 appears to be absent in the media for p67HA, but is present in the lysate (Figure 2b). As p67HA has a functional TM, it is possible that the 140 kDa protein is the form present at the cell surface. Why full-length p67 does not anchor to the plasma membranes of various cell lines is currently unknown. Wildtype p67 can be found on the surface of sporozoites; therefore, there may be a tick- or parasite-specific component required for anchorage that is absent in other cell types [45]. It is also possible that p67 may be unstable or processed in such a way that renders the native TM non-functional in heterologous expression systems. However, this is all speculative.

In addition to cell surface localisation, we have shown for the first time that p67 can be displayed on Gag-based VLPs through modification of the antigen, as observed with p67HA labelled by immunogold on the surface of both BLV Gag and HIV Gag VLPs (Figure 4). p67 with the native TM and CT could not be detected, which was not surprising, as Gag VLPs are enveloped and therefore require antigens at the plasma membrane for incorporation [32]. The choice of anchor for chimeric p67 was limited as not all TM and CT domains of other proteins can associate with Gag VLPs and they can differ in the density of antigen displayed [34]. The HA_2_ TM and CT was chosen as the anchor for p67 due to its ability to retain the incorporation of chimeric HIV envelope glycoprotein (Env) into HIV Gag VLPs [34] and the probability of a high density of accumulation. This anchor can be used across different retrovirus Gag VLPs and displayed antigens: the *Plasmodium falciparum* VAR2CSA antigen fused to HA_2_ TM and CT can incorporate into HIV Gag VLPs, and full HA can associate with murine leukaemia virus (MLV) Gag VLPs [46,47]. We further demonstrate its versatility by its ability to incorporate chimeric p67 into BLV Gag VLPs (Figure 4).

Encouragingly, the DNA plasmids encoding these proteins are immunogenic in vivo. Mice inoculated with single plasmids or plasmid combinations produced p67-binding antibodies, with titres ranging between 1:2430 to 1:7290 for pMExT p67, 1:240 to 1:21,780 for pMExT p67HA, 1: 2430 to 1:21,780 for pMExT p67HA + pTJDNA4 (HIV Gag) and all were 1:7290 for pMExT p67HA + pMEx BLV Gag (Figure 6b). There were no significant differences between any of the groups. The pMExT p67HA + pMEx BLV gag combination also elicited BLV Gag-binding antibodies, with titres ranging between 1:2430 to 1:65,610 (Figure 6c). Bovine leukaemia is endemic and is a problem in most parts of Africa where eradication by elimination of infected animals is not an option [48]. In HIV-infected individuals, responses to Gag are associated with control via CD8 (+) T-cells at the site of infection, control of spread from the entry portal, and control of viremia if infection is established [49]. Therefore, it is possible that a good immune response against BLV Gag may be able to act prophylactically and therapeutically, but this remains to be demonstrated. The plasmid backbone pMEx/pTHpCapR used for these constructs is known to induce high levels of expression and to significantly improve immune responses of the encoded antigens in mouse models [50]. This DNA vaccine backbone has not been tested in cattle. These high responses indicate that a dose sparing experiment should be performed to determine if the dose could be lowered in the target animals.

DNA vaccines circumvent the use of live *T. parva* parasites and thereby overcome some of the logistical issues associated with the current infection and treatment method against ECF [10]. A major advantage of supercoiled plasmid DNA is its stability; unlike *T. parva* sporozoites, DNA plasmids can be lyophilized and do not require liquid nitrogen for storage. However, the use of DNA as vaccines against various cattle diseases has been discouraging, due to their inability to provide adequate protection [51,52]. DNA vaccines appear more effective in cattle if used in DNA prime/protein boost regimes [53], therefore a similar approach might be more suitable for the antigens in this study. Moreover, the enhanced DNA expression vector pMEx/pTHpCapR would almost certainly significantly improve antigen expression in cattle, as it does in mice. Other platforms could also potentially be used for p67HA–VLP-based cattle vaccines, such as purified VLPs or live viral vectors instead of DNA, or a combination of platforms with a heterologous prime-boost strategy [35,40]. Additionally, the p67HA–VLP system could be combined into one plasmid encoding for both p67HA and Gag.

In conclusion, the DNA plasmids in this study provide a proof of concept that it is possible to display a form of *Theileria parva* p67 containing all known antigenic regions on the surface of Gag VLPs through modification of the C-terminal end. We show that both HIV and BLV Gag VLPs can be used as a nanoparticle for chimeric antigen display, which may be advantageous in the latter case due to their immunological relevance in cattle. Further investigation of the p67HA–Gag-VLP system is required to assess its use as a potential vaccine candidate against ECF.

## Supplementary Materials

The following are available online at https://www.mdpi.com/article/10.3390/vaccines10020210/s1, **Figure S1.** Purification of p67ΔTM from HEK293T cells (**a**) and BLV Gag from *E. coli* (**b**). Samples taken before (cell media and cell lysate), during (flow-through, wash 1 and wash 2) and after (eluate and concentrated eluate) purification of His-tagged p67ΔTM and BLV Gag through a cobalt-agarose column were used for SDS PAGE and Coomassie-blue staining. (**c**) Purified p67ΔTM was diluted in TBS from 1:5 up to 1:500 and subjected to SDS PAGE and Coomassie-blue staining as undiluted purified sample results in overloading, as also seen in (**a**).
