# Supplementary material for: Characterization of a Novel Chimeric Theileria parva p67 Antigen Which Incorporates into Virus-like Particles and Is Highly Immunogenic in Mice
Source: Vaccines (Basel). 2022 Jan 28;10(2):210. doi: 10.3390/vaccines10020210 (PMC8880696; doi:10.3390/vaccines10020210)
Supplement: Supplementary file 1 [file vaccines-10-00210-s001.zip › vaccines-1536182-supplementary.pdf]

Supplementary Materials

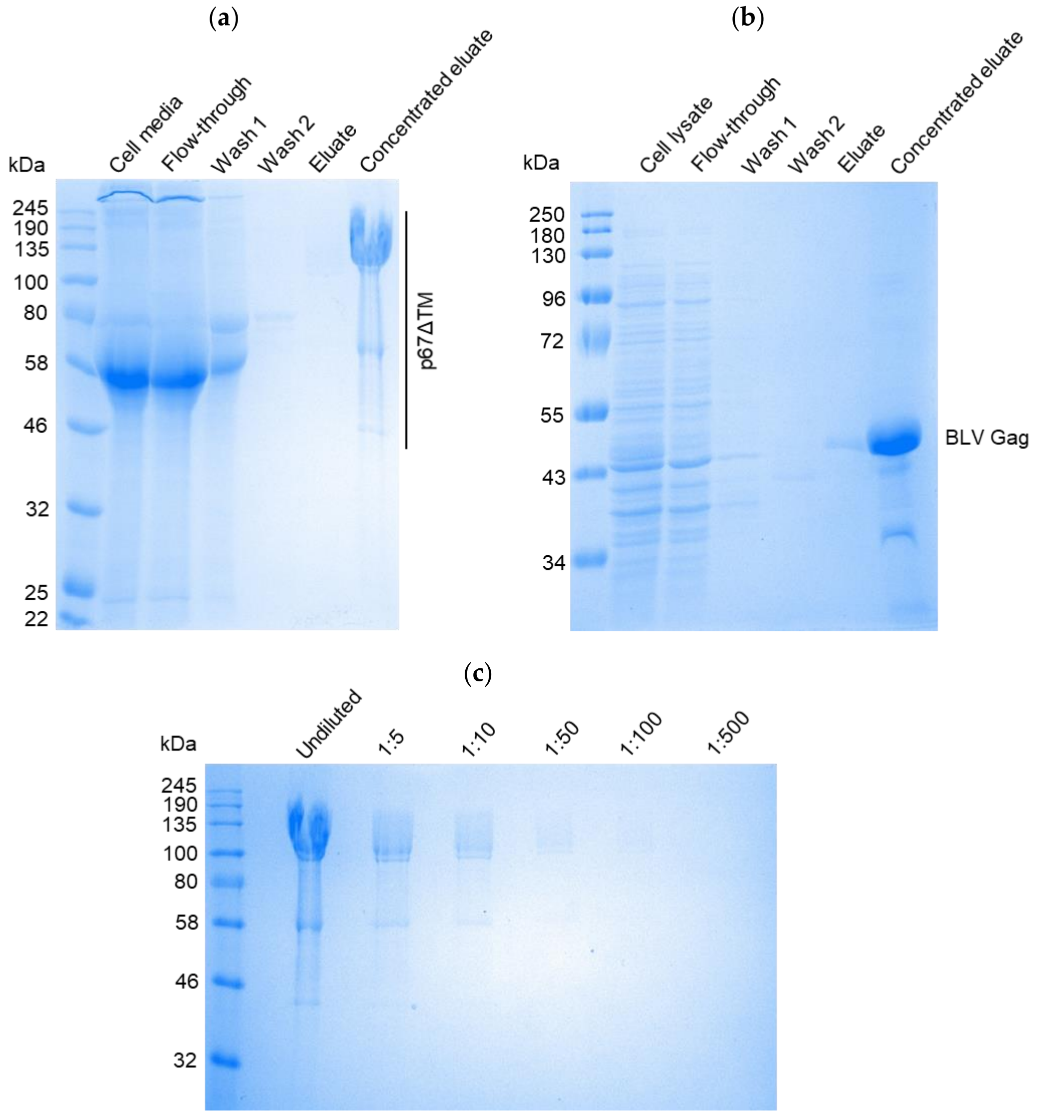

Figure S1. Purification of p67ΔTM from HEK293T cells (**a**) and BLV Gag from *E. coli* (**b**). Samples taken before (cell media and cell lysate), during (flow-through, wash 1 and wash 2) and after (eluate and concentrated eluate) purification of His-tagged p67ΔTM and BLV Gag through a cobalt-agarose column were used for SDS PAGE and Coomassie-blue staining. (**c**) Purified p67ΔTM was diluted in TBS from 1:5 up to 1:500 and subjected to SDS PAGE and Coomassie-blue staining as undiluted purified sample results in overloading, as also seen in (**a**).
